# Quarterly Report of Proceedings

**Published:** 1857-10

**Authors:** 


### QUARTERLY REPORT OF THE PROCEEDINGS.

*Monday, July 6th.* Dr. Snow in the Chair. "On the History of Gaol Fever in England." By Francis C. Webb, M.D., F.S.A. A discussion followed, in which Dr. Seaton, Mr. Lord, Dr. Camps, Dr. Richardson, Dr. Green, Mr. Hunt, and Dr. McWilliam, took part.

*Monday, August 30th.* A Paper was read by Dr. McWilliam on "The Local Causes Influencing the Diffusion and Type of Epidemics." By W. I. Cox, M.R.C.S. Dr. Tripe, Dr. Richardson, Dr. Camps, Mr. Burge, Dr. Babington, and Dr. McWilliam, took part in the discussion.

### PRESENTATIONS.

Several Books have been presented to the Society during the past quarter: the list is, however, deferred for want of room.

### NEW MEMBERS.

*Corresponding.* Jose Fernandez da Silva Leaõ, Surgeon-Major of the Cape de Verd Archipelago.

### NOTICE OF MEETING.

The opening Meeting of the ensuing Session of the Society will be held on Monday, November 2nd, when a Paper will be read by Dr. E. Headlam Greenhow.

